# Low-Level Laser Therapy and Photobiomodulation for Tinnitus and Sudden Sensorineural Hearing Loss: A Systematic Review

**DOI:** 10.7759/cureus.96234

**Published:** 2025-11-06

**Authors:** Hussain M Abdali, Lama B Almutairi, Muath H Alqesair, Layal M Alyala, Rawan N Omar, Ahmed A Altalhi, Najwa O Zakaria, Rahaf M Alhindi, Mohammed A Alkhurais, Abduallah A Mawkili

**Affiliations:** 1 College of Medicine, Jazan University, Jazan, SAU; 2 College of Medicine, Qassim University, Qassim, SAU; 3 Medical School, University of Szeged Albert Szent-Györgyi, Szeged, HUN; 4 College of Medicine, Taif University, Taif, SAU; 5 Otorhinolaryngology, Aseer Health Cluster, Abha, SAU; 6 Faculty of Medicine, University of Tabuk, Tabuk, SAU; 7 Nursing, Royal Commission Hospital, Yanbu, SAU; 8 College of Medicine, Batterjee Medical College, Jeddah, SAU

**Keywords:** audiometry, cochlear microcirculation, hearing rehabilitation, low-level laser therapy, photobiomodulation, sudden sensorineural hearing loss, systematic review, tinnitus

## Abstract

Tinnitus and sudden sensorineural hearing loss (SSNHL) are prevalent otologic disorders that can severely affect quality of life. Low-level laser therapy (LLLT) and photobiomodulation (PBM) have been proposed as potential treatments aimed at enhancing cochlear microcirculation and cellular metabolism; however, their clinical efficacy remains uncertain. This systematic review evaluated the effectiveness of LLLT and PBM in improving tinnitus severity and hearing outcomes among patients with tinnitus and SSNHL. A comprehensive search was conducted in PubMed, Scopus, Web of Science, and the Cochrane Library from inception to September 2025. Eligible studies included randomized controlled trials, quasi-experimental designs, and pre-post studies in humans, published in English, that investigated LLLT or PBM for these conditions. Methodological quality was assessed using the Downs and Black 28-item checklist. Nine clinical studies met the inclusion criteria. Most demonstrated short-term reductions in tinnitus severity and handicap immediately following treatment, with several reporting superiority over placebo or control groups. However, these benefits often diminished after three to six months in longitudinal follow-ups. Audiometric improvements were mainly observed in participants with moderate hearing loss, whereas minimal changes occurred in severe cases. Across all studies, LLLT and PBM were reported to be safe and well-tolerated. Overall, LLLT and PBM appear to offer meaningful short-term relief of tinnitus symptoms and modest improvements in hearing thresholds for selected patients, although their long-term efficacy remains uncertain. Variability in treatment parameters and outcome measures underscores the need for standardized protocols and high-quality randomized trials to support evidence-based clinical recommendations.

## Introduction and background

Tinnitus and sudden sensorineural hearing loss (SSNHL) are among the most common and distressing auditory disorders, affecting millions worldwide. Tinnitus, the perception of sound without an external source, affects approximately 10-15% of adults, with 1-2% experiencing severe symptoms that significantly impair quality of life [1]. SSNHL, defined as a rapid hearing loss of at least 30 dB across three contiguous frequencies within 72 hours, has an annual incidence of 5-30 cases per 100,000 people [2]. Both conditions are frequently associated with emotional distress, anxiety, and sleep disturbances, posing a considerable public health burden.

The mechanisms underlying tinnitus and SSNHL are multifactorial and not fully understood. Proposed pathophysiological processes include cochlear hair cell damage, aberrant auditory cortical activity, and impaired cochlear microcirculation [1]. In SSNHL, vascular compromise, viral infection, and autoimmune reactions are potential contributors [2]. These overlapping mechanisms can lead to both sudden hearing loss and the onset or worsening of tinnitus, creating diagnostic and therapeutic challenges that highlight the need for interventions targeting both auditory dysfunction and underlying cellular pathology [2].

Current management strategies for tinnitus and SSNHL remain largely empirical and often yield inconsistent results. Common treatments include systemic or intratympanic corticosteroids, vasodilators, antioxidants, and sound therapy, though many patients experience only partial or temporary relief [1,3]. Behavioral and cognitive therapies may reduce tinnitus-related distress but do not address its physiological basis, while pharmacologic agents targeting neurotransmission or cochlear metabolism have shown limited efficacy. Consequently, there is increasing interest in alternative and adjunctive approaches that promote cochlear repair, neuroplasticity, and microcirculatory improvement [1].

Low-level laser therapy (LLLT), also known as photobiomodulation (PBM), has emerged as a promising noninvasive modality for auditory rehabilitation. Using red or near-infrared light, PBM enhances mitochondrial activity, increases adenosine triphosphate (ATP) production, and improves cellular oxygenation and microcirculation within cochlear tissues [2]. Experimental and clinical studies suggest that PBM may support cochlear hair cell recovery, stabilize neuronal signaling, and reduce tinnitus severity by modulating oxidative stress and inflammation. Its biological plausibility and noninvasive nature have driven growing interest in its clinical application in otolaryngology [3].

Despite this interest, evidence on LLLT and PBM remains limited and heterogeneous, with variations in laser parameters, treatment duration, application methods, and outcome measures. Previous reviews have often included mixed populations or lacked rigorous methodological evaluation. Therefore, this systematic review aims to synthesize and critically appraise the available clinical evidence on the efficacy of LLLT and PBM for tinnitus and SSNHL.

## Review

Methodology

Literature Search Strategy

This systematic review followed the Preferred Reporting Items for Systematic Reviews and Meta-Analyses (PRISMA) guidelines [4]. A comprehensive search was conducted in PubMed, Web of Science, Scopus, and the Cochrane Central Register of Controlled Trials (CENTRAL) for studies published from inception to September 2025. The goal was to identify clinical studies of LLLT or PBM for tinnitus and SSNHL.

Search terms included low-level laser therapy, laser therapy, photobiomodulation, PBM, soft laser, cold laser, laser irradiation, laser phototherapy, tinnitus, subjective tinnitus, chronic tinnitus, sensorineural tinnitus, hearing loss, sudden sensorineural hearing loss, SSNHL, SNHL, cochlear dysfunction, and sensorineural deafness. Boolean operators (AND/OR) were used, and searches were limited to English-language studies in humans. Reference lists of included articles and relevant reviews were manually screened to identify additional studies.

Eligibility Criteria

Eligibility criteria were defined according to the PICO framework (Population, Intervention, Comparison, Outcome, Study design) [5]. Studies were included if they involved patients with tinnitus or sensorineural hearing loss (including SSNHL) treated with LLLT or PBM, regardless of wavelength, power, or duration. Comparators included placebo, sham laser, standard therapy, or pre-post self-comparison. Outcomes had to include at least one quantitative measure of tinnitus severity (e.g., Tinnitus Handicap Inventory (THI), Visual Analog Scale (VAS), Tinnitus Severity Index (TSI)) or hearing improvement (e.g., Pure Tone Audiometry (PTA)).

Only randomized controlled trials, quasi-experimental, and pre-post clinical studies published in English were included. Exclusion criteria comprised observational studies, case reports, reviews, animal or in vitro experiments, conference abstracts, unavailable full texts, or interventions unrelated to laser therapy.

Study Selection

**Figure 1:**
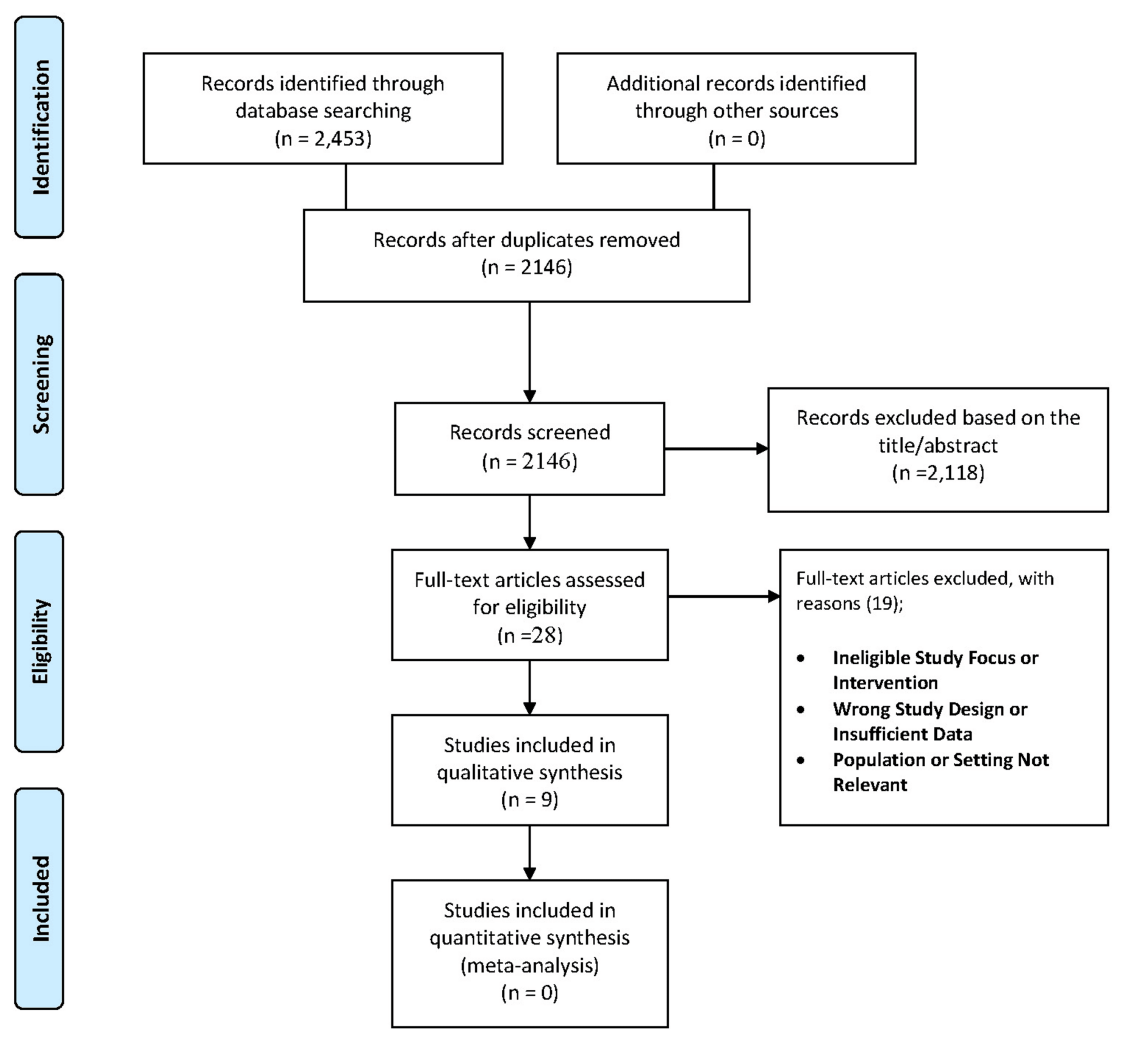
PRISMA flowchart for identification and inclusion of studies PRISMA: Preferred Reporting Items for Systematic Reviews and Meta-Analyses.

Two reviewers independently screened titles and abstracts using the eligibility criteria. Full texts of potentially relevant studies were reviewed for final inclusion. Disagreements were resolved through discussion or consultation with a third reviewer. After duplicate removal, 2,146 records were screened, 28 full texts assessed, and nine studies met the inclusion criteria [1-3,6-11]. The selection process is summarized in the PRISMA flow diagram (Figure 1).

Data Extraction

Data were extracted independently by two reviewers using a standardized form. Extracted variables included study ID, country, design, participant characteristics (sample size, age, gender, diagnosis, duration, severity), intervention details (laser wavelength, power, energy density, application site, exposure duration, and number of sessions), comparators, and outcome measures. Discrepancies were resolved through consensus or third-party review.

Quality Appraisal

Methodological quality was assessed independently by two reviewers using the Downs and Black 28-item checklist [12]. This tool evaluates five domains: reporting (10 points), external validity (3), internal validity-bias (7), internal validity-confounding (6), and power (2). Studies were rated as excellent (26-28), good (20-25), fair (15-19), or poor (<15). Disagreements were resolved through discussion until consensus was reached.

Results

*Study Selec*tion

A total of 2,453 records were identified from PubMed (n = 151), Cochrane Library (n = 57), Scopus (n = 987), and Web of Science (n = 1,258). After removing duplicates, 2,146 unique records were screened. Based on title and abstract review, 2,118 studies were excluded for irrelevance or ineligible design. Twenty-eight full-text articles were assessed, with 19 excluded for unsuitable interventions, designs, or populations. Nine studies met the inclusion criteria and were included in the qualitative synthesis. None were suitable for meta-analysis due to methodological heterogeneity and incomplete data.

Study Characteristics

The included studies varied in design and clinical setting, investigating LLLT and PBM for tinnitus and sensorineural hearing loss across Egypt, Turkey, Iran, Brazil, Iraq, and Malaysia. Participants were adults or adolescents with chronic subjective tinnitus, noise-induced hearing loss, or idiopathic and refractory tinnitus. Sample sizes ranged from 16 to 120 participants with a balanced gender distribution (Table 1).

**Table 1:** Summary of study characteristics LLLT: low-level laser therapy, PBM: photobiomodulation, RCT: randomized controlled trial, SNHL: sensorineural hearing loss, VAS: Visual Analogue Scale, THI: Tinnitus Handicap Inventory, TSI: Tinnitus Severity Index, PTA: pure tone audiometry, dB: decibel, Hz: hertz, nm: nanometer, mW: milliwatt, J/cm²: joules per square centimetre, μs: microsecond, LTE15: lasting therapeutic effect at 15 days, MRI: magnetic resonance imaging, TLLS: transmeatal low-power laser stimulation, Nd:YAG: neodymium-doped yttrium aluminium garnet.

Study ID (author, year)	Country	Study design	Population characteristics (sample size, age, gender, diagnosis, duration, severity)	Intervention details (LLLT/PBM parameters: wavelength, power, energy density, site, duration, sessions)	Comparison/control	Outcome measures (primary and secondary)	Main findings
Demirkol et al., 2017 [1]	Turkey	Prospective, randomized, placebo-controlled, single-blind trial	46 patients (23 males, 23 females; aged 13–65 years) with bilateral subjective tinnitus associated with temporomandibular disorders (≥6 months). Patients with hearing loss, Ménière’s disease, or otitis were excluded.	Three groups: (1) Nd:YAG laser (1064 nm, 0.25 W, 8 J/cm², 1000 μs, 10 Hz, 20 s/ear, 5 days/week × 10); (2) Diode laser (810 nm, 0.25 W, 8 J/cm², 9 s/ear, same schedule); (3) Placebo (no emission). Probe applied parallel to the external auditory canal.	Placebo laser identical to intervention (no emission).	Primary: VAS for tinnitus loudness (baseline and 1 month post-treatment).	Significant improvement in VAS for Nd:YAG (median 5 → 0; p = 0.001) and diode laser (8 → 5.5; p = 0.005). No change in placebo (p = 0.065). Percent improvement: Nd:YAG 100%, diode 30%, placebo 0%. No adverse effects.
Toson et al., 2016 [2]	Egypt	Randomized controlled trial (RCT)	60 patients (30 per group), aged 30–50 years; 50% male/female. All had chronic subjective tinnitus (≥6 months), unilateral or bilateral, with or without SNHL.	Infrared LLLT (904 nm); 20 min/session; 3 sessions/week for 1 month; probe placed on mastoid process; affected ear treated (worse ear in bilateral cases).	Placebo laser (identical parameters, no emission).	Primary: VAS for tinnitus loudness; Tinnitus Severity Index (TSI).	Significant post-treatment reduction in VAS (7.2 ± 1.73 → 3.2 ± 2.68; p = 0.0001) and TSI (40 → 25; p = 0.0001). Control group showed minimal change. Between-group differences favored LLLT (p < 0.05). No adverse effects reported.
Panhóca et al., 2023 [3]	Brazil	Double-blind, randomized controlled clinical trial	107 patients (57 males, 50 females; mean age 49.9 ± 8.2 years) with idiopathic or refractory tinnitus. Excluded: diabetes, hypertension, obesity, cancer.	Ten groups (n = 10–11): G1 (control, device off); G2 (vacuum therapy + transcochlear LLLT 660 + 808 nm, 100 mW, 3 min, −120 mbar); G3 (G2 + flunarizine 10 mg/day); G4 (flunarizine only); G5 (LLLT 660 + 808 nm + ultrasound 1 MHz, 1 W/cm², 50% duty, 180 s); G6 (transmeatal LLLT 660 nm, 100 mW, 6 min); G7 (G6 + Ginkgo biloba 120 mg/day); G8 (Ginkgo only); G9 (transmeatal LLLT 660 nm, 100 mW, 15 min); G10 (laser puncture, 4 J, 23 acupoints). All: 2 sessions/week × 4 weeks (8 sessions).	G1 (control, device off).	Primary: THI (pre-, post-, 15 days post-treatment). Secondary: 15-day lasting therapeutic effect (LTE15).	Significant improvement: G9 (15 min LLLT) − THI ↓ 56.4%; G10 (laser puncture) − 44.7%; G2 (vacuum + LLLT) − 41%. Shorter LLLT (6 min) less effective (+39.6% gain with 15 min). G9/G10 maintained effect at 15 days. No adverse effects.
Aldergazly & Khlaif, 2018 [6]	Iraq	Experimental pre–post study (two treatment protocols, no control group)	16 patients (30 ears), aged 9–18 years, with SNHL (40–75 dB; moderate–severe).	Two diode lasers (red 650 nm + green 532 nm) via a fiber coupler. Group 1: 8 patients (14 ears), 20 mW, 2.78 mW/cm², 30 min/day (15 min mastoid + 15 min ear canal) × 10 days (5.004 J/cm²). Group 2: 8 patients (16 ears), same settings, 40 min/day (20 min each site) × 5 days (6.672 J/cm²).	None (pre–post comparison).	Primary: Pure Tone Audiometry (PTA) thresholds (500 Hz–2 kHz).	Significant PTA improvement in moderate/moderately severe cases (58.6 ± 11.5 → 44.8 ± 9.6 dB; p = 0.0001; 23.6% gain). No change in severe loss subgroup. Temporary benefit, more evident in moderate cases.
Masry et al., 2021 [7]	Egypt	Randomized, placebo-controlled, double-blind clinical study	30 patients (16 males, 14 females; mean age 36.3 ± 12.4 years, range 18–55) with bilateral SNHL and bilateral subjective tinnitus.	LLLT applied to one ear and placebo to the other. Device: Tinnitool (Dismark GmbH, Switzerland); 650 nm, 5 mW, 15 min/session, 3 sessions/week × 7 weeks (21 sessions). Delivered transmeatally via fiber-optic headband.	Placebo laser (same setup, inactive) to contralateral ear.	Primary: VAS and THI before, 3 months, and 6 months post-treatment. Secondary: THI grades.	3 months: VAS 8 → 5.5 (p = 0.013), THI 54.7 → 38.1 (p < 0.0001). 6 months: effect partially lost. THI grade 4 decreased from 46.7% to 6.7% at 3 months, rose to 13.3% at 6 months. No adverse events.
Mirvakili et al., 2014 [8]	Iran	Randomized controlled trial	120 patients (60 per group; mean age 39.8 ± 5.5 years; 50% male) with intractable tinnitus secondary to SNHL (>1 year, medication-refractory).	LLLT (TINNImed, Switzerland): 650 nm, 5 mW; transmeatal via silicon probe targeting cochlea; 20 sessions (20 min, 3 sessions/week).	Placebo device (inactive laser).	Primary: VAS (0–10) and THI (25 items). Secondary: ≥2-point VAS improvement; follow-up at 3 months.	End of treatment: VAS 5.69 → 4.28 vs. 6.46 → 6.27 (control); p = 0.001. THI 3.01 → 1.93 vs. 2.73 → 2.35; p = 0.01. At 3 months: no significant difference (p = 0.85). VAS ≥2-point improvement: 56% LLLT vs. 30% control (p = 0.003). No adverse effects.
Mollasadeghi et al., 2013 [9]	Iran	Double-blind randomized clinical trial	89 male industrial workers (aged 30–51 years; mean 41.17 ± 5.89) with tinnitus due to noise-induced hearing loss (mean duration 1.85 ± 0.78 years; 49% bilateral).	LLLT (TINNImed, Switzerland): 650 nm, 5 mW, 20 min/session every other day × 20 session; applied via mastoid soft silicone tip.	Placebo device (laser off).	Primary: VAS, THI, and tinnitus loudness (dB). Secondary: ≥50% reduction in VAS/THI.	Post-treatment significant improvement in LLLT vs. placebo (p < 0.001). ≥50% VAS reduction: 29% vs. 7.5%; ≥50% THI reduction: 43% vs. 10%. At 3 months: partial loss but still significant (p = 0.009). No adverse effects.
Ngao et al., 2013 [10]	Malaysia	Double-blind, randomized, placebo-controlled clinical trial	43 patients (26 females, 17 males; mean age 57.6 years, range 40–70) with persistent subjective tinnitus; retrocochlear pathology excluded by MRI and audiometry.	Transmeatal Low-Power Laser Stimulation (TLLS): MedicLaser + Tinnitool (Dismark GmbH, Switzerland); 650 nm, 5 mW; 20 min/day for 10 weeks via fiber-optic headband. All patients also received betahistine 24 mg twice daily for 10 weeks.	Placebo device (inactive), same betahistine regimen.	Primary: THI and VAS for annoyance, sleep, depression, concentration, loudness, and pitch. Secondary: THI grades (McCombe classification).	Both groups improved in THI and VAS within groups (p < 0.05) but not between groups (p = 0.353). THI grade improvement: 36% (TLLS⁺) vs. 57% (TLLS⁻); p = 0.405. No hearing deterioration or adverse effects.
Okhovat et al., 2011 [11]	Iran	Self-controlled clinical trial	61 patients (38 males, 23 females; mean age 40.5 ± 15.3 years) with chronic subjective tinnitus (>6 months), unilateral or bilateral. 14 (31.8%) were exposed to occupational noise.	LLLT (TINNImed soft laser): 650 nm, 5 mW, continuous beam; transmeatal via silicone probe directed at tympanic membrane/cochlea; 20 min/day × 20 consecutive days.	None (self-controlled pre–post comparison).	Primary: VAS (0–100%) for tinnitus loudness. Secondary: subgroup analysis by age, gender, and noise exposure.	Mean VAS reduction 82.3 ± 18.3% → 35.9 ± 38.7% (p < 0.0001). Complete resolution in 18%, partial in 49%, no change in 33%. Greater improvement in younger patients (p < 0.05). Noise exposure was significant in men (p = 0.029). No adverse effects.

Most trials applied transmeatal irradiation targeting the cochlea, using wavelengths between 650 and 660 nm with power outputs of 5-100 mW. Others used mastoid or combined mastoid-canal applications [7-10]. Treatment duration ranged from 6 to 20 minutes per session, two to five times per week for 8-20 sessions. Some studies used infrared 904 nm [13], Nd:YAG 1064 nm or diode 810 nm [1], and one combined red (650 nm) with green (532 nm) lasers [6].

Control conditions were usually sham lasers with identical appearance but inactive emission, ensuring blinding. A few trials used pre-post self-comparisons without external controls [6,11]. Primary outcome measures included the THI and VAS, supplemented in some studies by the TSI and PTA [3]. Secondary outcomes included quality-of-life scores, residual inhibition, and follow-up response durability at three to six months. Collectively, these studies demonstrated heterogeneity in parameters but provided complementary evidence on wavelength, duration, and delivery effects on LLLT/PBM efficacy [3,7,8].

Quality Assessment

Using the Downs and Black 28-item checklist, most studies achieved good methodological quality (scores 20-25/28). The highest ratings were seen in trials by Panhóca et al. [3] and Mollasadeghi et al. [9], both scoring 25/28, with strong reporting and internal validity. Demirkol et al. [1], Masry et al. [7], and Ngao et al. [10] scored 24/28, reflecting robust blinding and detailed protocols. Toson et al. [2], Mirvakili et al. [8], and Okhovat et al. [11] scored 20-21/28, indicating moderate quality with minor reporting gaps (Table 2).

**Table 2:** Quality assessment of included studies Scores represent the number of criteria fulfilled within each domain of the Modified Downs and Black checklist [12]. Values in parentheses indicate the maximum possible score for that category: reporting (10), external validity (3), internal validity – bias (7), internal validity – confounding (6), and power (2). Higher total scores reflect stronger methodological quality.

Study ID	Reporting (10)	External validity (3)	Internal validity – bias (7)	Internal validity – confounding (6)	Power (2)	Total (28)
Demirkol et al. [1]	9	3	6	5	1	24/28
Toson et al. [2]	9	2	5	4	0	20/28
Panhóca et al. [3]	10	3	6	5	1	25/28
Aldergazly & Khlaif [6]	7	2	4	3	0	16/28
Masry et al. [7]	9	3	6	5	1	24/28
Mirvakili et al. [8]	9	2	5	4	0	20/28
Mollasadeghi et al. [9]	10	3	6	5	1	25/28
Ngao et al. [10]	9	3	6	5	1	24/28
Okhovat et al. [11]	9	2	5	4	1	21/28

Aldergazly and Khlaif [6] achieved a score of 16/28, rated fair due to lack of blinding and control, but provided valuable pediatric data. Overall, reporting and internal validity were strong, while external validity was moderate (scores 2-3). Power analyses were rarely reported, underscoring the need for larger, standardized multicenter trials.

Overall Trends of Interventions

Across transmeatal, auriculotherapy, and temporomandibular-focused protocols, most studies showed short-term reductions in tinnitus severity, greatest immediately post-treatment or within one to three months [1,2,8,9]. Several reported greater improvement compared with control, suggesting true treatment effects [1,2,8]. Benefits typically waned by three to six months [7,8], highlighting limited durability. Longer exposure times and auriculotherapy protocols yielded the largest short-term benefits [3].

Effect of Interventions on Tinnitus Severity

VAS reductions were consistent across active treatment groups. Notable improvements included approximately 56% reduction in active versus 12% in control in Toson et al. [2]; significant baseline-to-post reductions in Nd:YAG and diode groups in Demirkol et al. [1]; and higher responder proportions (≥2-point improvement) in Mirvakili et al. [8]. Occupational cohorts also showed immediate relief, though response variability was high [9]. In uncontrolled data, the average VAS reduction reached 36%, with better outcomes in younger participants and reduced benefit in noisy workplaces [11]. Most gains diminished by three to six months, as seen in Masry et al. [7], where mean VAS improved from 8.0 to 5.5 at three months but returned to 7.5 by six months.

Effect of Interventions on Tinnitus Handicap

Handicap scores mirrored VAS trends. THI and TSI improved more in active than in control groups [3,8], with notable short-term THI reductions (mean 54.7 to 38.1) that partially relapsed by six months [7]. The largest THI improvements were reported with 15-minute auriculotherapy and laser puncture compared with shorter exposure protocols [3], underscoring time sensitivity.

Effect of Interventions on Hearing Thresholds

Audiometric results were mixed and condition-dependent. Pediatric and adolescent participants with moderate hearing loss improved from approximately 58.6 to 44.8 dB using combined red/green lasers [6], while severe loss showed minimal recovery. In adults, symptomatic relief was often unaccompanied by audiometric change [8-10], indicating dissociation between tinnitus perception and threshold recovery.

Effect of Interventions on Placebo-Controlled Outcomes and Heterogeneity

Controlled studies generally favored active LLLT in the short term. Significant within-group improvements were limited to active arms [1,8,9], although some reported similar THI gains in placebo groups, reflecting strong nonspecific effects [10]. Longer exposure and laser puncture yielded greater improvements after adjustment for placebo response, while certain adjunctive combinations offered limited additional benefit [3].

Durability of Interventions

Therapeutic effects commonly declined by three to six months without maintenance therapy. Early post-treatment gains often diminished by follow-up [8,13]. However, short-term persistence (up to 15 days) was noted in regimens with longer exposure or laser puncture, suggesting potential for booster sessions to sustain benefit [3].

Moderators and Subgroups of Interventions

Younger age correlated with greater improvement, while noisy occupational environments reduced response [11]. Treatment parameters, such as laser type, site, and duration, strongly influenced effect size: Nd:YAG and diode both improved VAS scores [1], and extended auricular exposure enhanced THI outcomes [3]. Efficacy also extended to tinnitus secondary to temporomandibular disorders [1].

Safety and Adherence of Interventions

LLLT was consistently safe and well-tolerated, with no serious adverse events reported [8-11]. Adherence was good, and attrition rates were low to moderate, with no differential dropout between groups [9,10].

Summary of Findings

LLLT and PBM yield clinically meaningful short-term reductions in tinnitus loudness and handicap, often outperforming placebo at the end of therapy [1,3,8]. Audiometric gains are inconsistent and appear limited to moderate hearing loss in younger patients [6,10]. The main limitation is the transient nature of improvement, typically waning by three to six months [7,8]. Optimized dosing, particularly extended auriculotherapy and laser puncture, enhances early efficacy and may prolong benefit [3]. Future studies should employ standardized protocols, rigorous sham controls, subgroup analyses, and long-term follow-up to confirm durable therapeutic value.

## Conclusions

LLLT and PBM demonstrate promising short-term benefits in reducing tinnitus severity and perceived handicap, but these effects often diminish within three to six months, highlighting limited long-term efficacy. Treatment outcomes appear to be influenced by irradiation parameters such as wavelength, exposure duration, and target site, with auricular, transmeatal, or laser puncture techniques showing more consistent results. Both modalities are safe and well tolerated, although audiometric gains in sensorineural hearing loss remain modest, suggesting that symptom relief likely arises from neuromodulatory rather than structural mechanisms. Given the heterogeneity and methodological limitations of existing studies, large, standardized, and long-term randomized trials are needed to validate efficacy and to define optimal therapeutic protocols.
